# Unlocking timely palliative care: assessing referral practices and barriers at a ghanaian teaching hospital

**DOI:** 10.1186/s12904-024-01411-9

**Published:** 2024-04-05

**Authors:** Tabitha Gyanewaa Quaidoo, Barbara Adu, Merri Iddrisu, Frema Osei-Tutu, Candace Baaba, Yekua Quiadoo, Collins Atta Poku

**Affiliations:** 1 Department of Surgery, Palliative Care Unit, Korle Bu Teaching Hospital, Accra, Ghana; 2 Department of Obstetrics and Gynaecology, Korle Bu Teaching Hospital, Accra, Ghana; 3 School of Nursing and Midwifery, University of Ghana, Legon, Ghana; 4 Ga East Municipal Hospital, Accra, Ghana; 5 Department of Humanity, University of Ghana, Legon, Ghana; 6 School of Nursing and Midwifery, Kwame Nkrumah University of Science and Technology, Kumasi, Ghana

**Keywords:** Palliative care, Referral practices, Perceived barriers, Physicians

## Abstract

**Background:**

The need for primary care physicians to be heavily involved in the provision of palliative care is growing. International agencies and practice standards advocate for early palliative care and the use of specialized palliative care services for patients with life-threatening illnesses. This study was conducted to investigate physicians’ referral practices and perceived barriers to timely referral at the Korle Bu Teaching Hospital.

**Methods:**

A cross-sectional study design was employed using a convenience sampling technique to recruit 153 physicians for the study. Data on socio-demography, referral practices, timing and perceived barriers were collected using a structured questionnaire. Binary Logistic regression using crude and adjusted odds was performed to determine the factors associated with late referral. Significance was set at *p* < 0.05.

**Results:**

The prevalence of late referral was reported to be 68.0%. There were poor referral practices among physicians to palliative care services, and the major barriers to late referral were attributed to the perception that referring to a palliative care specialist means that the physician has abandoned his patient and family members’ decisions and physicians’ personnel choices or opinions on palliative care.

**Conclusion:**

The healthcare system needs tailored interventions targeted at improving physicians’ knowledge and communication strategies, as well as tackling systemic deficiencies to facilitate early and appropriate palliative care referrals. It is recommended that educational programs be implemented, palliative care training be integrated into medical curricula and culturally sensitive approaches be developed to address misconceptions surrounding end-of-life care.

**Supplementary Information:**

The online version contains supplementary material available at 10.1186/s12904-024-01411-9.

## Background

Globally, the burden of chronic and life-limiting illnesses is on the rise [1, 2]. As projected by the World Health Organization (WHO) [3], nearly 74 per cent of all deaths worldwide will be caused by non-communicable diseases (NCDs) by 2025, 85% of them occurring in low- and middle-income countries (LMICs). NCDs are predicted to cause 41 million deaths in LMICs by 2025, mostly from cancers (21%), cardiovascular diseases (48%), diabetes (3%) and chronic respiratory diseases (12%), if proper prevention approaches are not designed and applied [4, 5]. With the increasing prevalence of chronic NCDs, the role of physicians has become limited in controlling and palliating symptoms leading to greater attention on palliative care [6, 7].

The WHO estimates that 40 million people require palliative care, with 78 per cent of those individuals residing in low- to LMICs [8]. To lessen suffering and enhance quality of life, palliative care comprises attending to the affected person’s physical, emotional, spiritual, social, and intellectual needs [9, 10]. Timely involvement and integration of palliative care into health delivery services have been associated with a positive quality of life and survival of patients [11, 12]. Concurrent palliative care from the time of diagnosis has been recommended for all patients with high disease burden with the 2014 World Health Assembly Resolution also recommending integrated palliative services into existing health systems [13, 14]. By 2020, more than two-thirds of the world’s nations provided hospice and palliative care, and 45 of those nations also included integrated palliative services into their larger healthcare systems [11]. Notwithstanding, palliative care integration remains challenging [15]. Historically, palliative care development in sub-Saharan Africa (SSA) has been very low compared to other parts of the continent [16]. However, it is significant to acknowledge the commendable efforts made by some countries, such as South Africa and Uganda, in advancing palliative care practices and initiatives in the region. These include training and provision of essential infrastructure and resources in ensuring the provision of palliative care services [17–19]. There is increasing demand for primary care practitioners to play a key role in palliative care delivery. Primary care practitioners can facilitate timely and appropriate access to specialized palliative care services, however, the highly individualized and idiosyncratic nature of decision-making around referral to palliative care emphasizes the need for research on how specialists communicate palliative care decisions [20].

In Ghana, there have been concerns about how physicians’ knowledge of palliative care impacts palliative care practice [21, 22]. Physicians have a leading role in promoting treatments that reduce suffering and improve the quality of life for terminally ill patients [23]. However, there are diverse variables that can impede or promote the referral process. It is, therefore, necessary that continuous research is conducted among physicians to support and educate them for the provision of high-quality palliative and end-of-life care [20, 24, 25].

The Korle Bu Teaching Hospital (KBTH), is a tertiary institution where formal palliative care is being given. Though most physicians and nurses who provide palliative care services are not trained specialists, palliative care is embedded in the respective curriculum for the training of the various cadre of the health workforce, and this gives these professionals the basic expertise in providing care. Also, in-service training on palliation is periodically provided to physicians and nurses. To aid in the smooth referral of cases, the department has a well-developed guideline that supports physicians on cases that will need referrals for palliative care services. Despite efforts by the institution to develop guidelines and training of healthcare providers to provide basic palliative care services to patients who may need them, most patients living with life-threatening conditions and end-stage conditions pass on without any palliative care. For the few who get access to palliative care services, this is done only when patients have become unresponsive to treatment. There is, therefore, the need to clearly understand the referral practices and perceived barriers to timely palliative care among physicians at the KBTH-Accra Ghana. The study’s findings will contribute to enhancing the timely and effective delivery of palliative care services at the KBTH in Ghana by identifying and addressing existing referral practices and overcoming barriers to access. The study will also contribute to the global understanding of perceived barriers to timely referrals by examining their impact within the context of palliative care services.

## Materials and methods

### Design, setting and participants

A cross-sectional design was used for the study. Data was collected between December 2021 and February 2022. The study was conducted at the KBTH. KBTH is a 2,000-bed tertiary care hospital located in Accra, Ghana. Among the many services offered by the KBTH are urology, neurosurgery, orthopaedics, general surgery, internal medicine etc. The hospital has a palliative care unit which has a system for identifying patients in need of palliative care and a protocol for referral. The palliative care staff provide care to in-patients in the department who may need their service. The participants were recruited from six [6] departments of the KBTH (Obstetrics and Gynaecology, Surgical, Medical, Child Health, Cardiothoracic and Haematology). The study comprised physicians who had spent at least a year working in any of the six KBTH departments and were involved with the referral of at least a palliative case to a specialist. The study excluded physicians who did not consent to be part of the study.

### Sample size and sampling

Slovin formula was used to estimate a sample size of 216 for the study. A convenience sampling technique was used in recruiting the respondents. To recruit respondents, the researchers first obtained ethical clearance from the hospital to conduct the study. The researchers then identified eligible respondents by reviewing the staff roster and meeting with the departmental heads. Eligible respondents were then approached by the researchers, who explained the purpose of the study and invited them to participate.

### Measure

Data was collected using self-administered questionnaires. The questionnaire was developed by the researchers through an extensive review of related literature and modified according to an instrument used in past surveys [20, 24, 26, 27]. The questionnaire sought to elicit responses from physicians who were involved in referrals of any case (including cancers, terminal stages of other non-communicable diseases, tuberculosis, AIDS etc.) for palliative care. The first section of the questionnaire consisted of questions that elicited information on demographic characteristics such as age, sex, department, number of years, rank and religion. The second section consisted of questions about barriers to referrals and individuals responsible for late referrals. It contained 10 items and was scored on response (Yes, No, Don’t). The questionnaire was revised to improve clarity and formatting after pre-testing 15 physicians at Ridge Hospital. The questionnaire is attached as a supplementary file.

### Data analysis

Data analysis was done using IBM SPSS version 26. Descriptive statistics was used to analyse the demographic variables and referral practices and were reported as proportions, percentages and frequencies. Continuous variables were analyzed using mean and standard deviation. The chi-square test of independence was used to establish cross-tabulation of counts and percentages. The association between sociodemographic factors, palliative care practices, timing of referrals and perceived barriers to early referral was analyzed using binary logistic regression. The odds ratio and adjusted odds ratio were used to determine the risk factors contributing to late referral practices. Statistical significance was set at a p-value of < 0.05.

### Ethical consideration

According to the Declaration of Helsinki, ethical approval was obtained from the institutional review board (IRB) of the Korle Bu Teaching Hospital (KBTH-STC 000181/2021). All methods were carried out in accordance with relevant guidelines and regulations. Respondents who agreed to participate provided written informed consent before the study. They were assured that their participation was voluntary, that their professional practice would not be affected by their decision to participate, and that their personal information would be kept confidential.

## Results

With a response rate of 70.8%, 153 questionnaires were included in the analysis. Table 1 details the demographic data of participants. While more than half of the respondents (50.7%) were below the age of 30 years, 62.7% were males. The majority of the respondents were Christians (91.5%) and had more than 3 years of practice (60.1%). Close to a third (30.7%) of the respondents worked in the surgical department with 38.8% being house officers. Late referrals due to clinician-related barriers were reported in 68.0% of cases, while 17.6% of cases were due to family and patient-related issues. Physicians’ perception of palliative care was reported as a reason for late referrals in 13.7% of cases.

**Table 1:** Personal and work characteristics of respondents

Variable	Frequency	Percentage
**Age group**		
≤ 30	79	51.6
31–45	52	34.0
46–50	12	7.8
> 50	10	6.6
**Gender**		
Male	96	62.7
Female	57	37.3
**Department**		
Obstetrics and Gynaecology	41	26.8
Surgical	47	30.7
Medical	20	13.1
Child health	10	6.5
Cardiothoracic	15	9.8
Haematology	20	13.1
**Rank**		
House officer	59	38.6
Medical Officer	18	11.8
Resident	55	35.9
Consultant	21	13.7
**Religions affiliation**		
Christianity	140	91.5
Islam	12	7.8
Other	1	0.7
**Years of practice**		
≤ 3	61	39.9
> 3	92	60.1
**Late referral due to physician-related barriers**		
Yes	104	68.0
No	49	32.0
**Late referral from family and patient-related issues**		
Yes	27	17.6
No	126	82.4
**Late referral due to physicians’ perception of palliative care**		
Yes	21	13.7
No	132	86.3

### Barriers to early referral for palliative care services

Table 2 details the barriers that lead to late referral of patients for palliative care. Though, the majority of respondents 68% perceived significant barriers to referring their patients to palliative care specialists, most of the respondents ascribed the phenomenon to the cause of the barrier. The most common was the perception that ‘referring a patient to a palliative care specialist meant that I abandoned my patient’ (92.8%). Also, the perception that ‘patients or family members did not like being referred to palliative care’, with 77.8% of respondents reporting this as a concern. Another commonly cited barrier was long-standing relationships with their patients (35.9%).

**Table 2:** Perceived barriers to late referrals to palliative care specialist

Perceived Barriers to Palliative Care Specialists	Yes	No	I don’t know
Palliative care specialists are not available in my hospital	12 (7.8)	130 (85.0)	11 (7.2)
Appointments with palliative care specialists are hard to get	19 (12.4)	120 (78.4)	14 (9.2)
My patients or family members do not like being referred to a palliative care specialist.	119 (77.8)	24 (15.7)	10 (6.5)
Referring to a palliative care specialist means that I abandon my patient	132 (92.8)	19 (12.4)	2 (1.3)
Palliative care specialists discourage active oncological therapy	9 (5.9)	135 (88.2)	9 (5.9)
Palliative care specialists in my country are not experienced/trained enough.	11 (7.2)	130 (85.0)	12 (7.8)
I can provide better symptom control management than them.	15 (9.8)	129 (84.3)	9 (5.9)
Patient deteriorates faster	26 (17.0)	112 (73.2)	15 (9.8)
Long-standing relationship with patient	55 (35.9)	89 (58.2)	9 (5.9)
Poor relationship with palliative care specialist	20 (13.1)	122 (79.7)	11 (7.2)

### Association between referral practices and independent variables

The association between referral practices and the demographic variables are detailed in Table 3. The results show the distribution of late and early referrals among 153 respondents in a tertiary hospital. The majority of physicians were males (59.4%) and affiliated with Christianity (84.3%). The departments with the highest rates of late referrals were surgical (27.9%) and obstetrics and gynaecology (24.0%). Late referrals were significantly associated with department, religious affiliation, years of practice, and late referral from family and patient-related issues (*p* < 0.05). Late referral due to physicians’ perception of palliative care was also significant (*p* < 0.01). However, age group, gender, rank, and late referral due to clinicians-related barriers were not significantly associated with late referrals (*p* > 0.05).

**Table 3:** Association between referral practices and independent variables

Variable	Late referral (n %)	Early referral (n %)	p-value
**Age group**			0.49
≤ 30	58 (55.8)	18 (36.7)	
31–45	32 (30.8)	17 (34.7)	
46–50	6 (5.8)	5 (10.2)	
> 50	8 (7.6)	9 (18.4)	
**Gender**			0.74
Male	57 (59.4)	39 (40.6)	
Female	36 (63.2)	21(36.8)	
**Department**			**0.001**
Obstetrics and Gynaecology	25 (24.0)	17 (34.7)	
Surgical	29 (27.9)	13 (26.5)	
Medical	14 (13.5)	9 (18.4)	
Child health	5 (4.8)	10 (20.4)	
Cardiothoracic	15 (14.4)	0 (0)	
Haematology	16 (15.4)	0 (0)	
**Rank**			**0.14**
House officer	39 (37.5)	20 (40.8)	
Medical Officer	11 (10.6)	12 (24.5)	
Resident	36 (34.6)	14 (28.6)	
Consultant	18 (17.3)	3 (6.1)	
**Religions affiliation**			**0.02**
Christianity	118 (84.3)	22 (15.7)	
Islam	5 (41.7)	7 (58.3)	
Other	1 (100)	0 (0)	
**Years of Practice**			**0.001**
≤ 3	38 (33.6)	23 (57.5)	
> 3	75 (66.4)	17 (42.5)	
**Late referral due to clinicians related barrier**			0.09
Yes	32 (27.1)	17 (42.5)	
No	81 (72.9)	23 (57.5)	
**Late referral from family and patient-related issues**			**0.04**
Yes	14 (12.3)	13 (32.5)	
No	99 (87.7)	27 (67.5)	
**Late referral due to physicians’ perception of palliative care**			**0.01**
Yes	11 (9.7)	10 (25.0)	
No	102 (90.3)	30 (75.0)	

### Factors associated with referral practices

Table 4 details the results of the factors that are associated with referral practices. It showed that the odds of late referral were not significantly different among age groups. However, participants aged 46–50 years had 63% increased odds of late referral compared to the reference group aged ≤ 30 years, although this finding was not statistically significant. Similarly, participants aged > 50 years had 41% decreased odds of late referral compared to the reference group, but this finding was also not statistically significant. Gender was not found to be significantly associated with late referral. Among the different departments, surgical department participants had 26% increased odds of late referral compared to obstetrics and gynaecology department participants, although this finding was not statistically significant. Late referral due to clinicians’ related barriers was significantly associated with reduced odds of late referral, while late referral due to family and patient-related issues and physicians’ perception of palliative care were both significantly associated with reduced odds of late referral in the crude odds ratios but did not remain significant in the adjusted odds ratios.

**Table 4:** Factors associated with referral practices

Variables	COR (95%CI)	AOR (95%CI)
Age group		
≤ 30	1.00 (ref)	1.00 (ref)
31–45	0.79 (0.39–1.61)	0.60 (0.26–1.41)
46–50	2.02 (0.64–6.36)	1.63 (0.45–5.91)
> 50	0.98 (0.27–3.54)	0.59 (0.13–2.62)
**Gender**		
Male	1.10 (0.61–1.98)	1.06 (0.54–2.10)
Female	1.00 (ref)	1.00 (ref)
**Department**		
Obstetrics and Gynaecology	1.00 (ref)	1.00 (ref)
Surgical	1.41 (0.75–2.63)	1.26 (0.62–2.56)
Medical	0.69 (0.29–1.65)	0.77 (0.30–1.99)
Child health	0.32 (0.08–1.29)	0.29 (0.06–1.42)
**Rank**		
House officer	1.00 (ref)	1.00 (ref)
Medical Officer	0.57 (0.23–1.44)	0.53 (0.19–1.47)
Resident	0.35 (0.16–0.75)	0.26 (0.11–0.62)
Consultant	0.80 (0.30–2.14)	0.72 (0.24–2.14)
**Religions affiliation**		
Christianity	1.00 (ref)	1.00 (ref)
Islam	0.42 (0.11–1.62)	0.51 (0.11–2.41)
Other	-	-
**Years of Practice**		
≤ 3	1.00 (ref)	1.00 (ref)
>3	2.05 (1.08–3.90)	2.61 (1.24–5.52)
Late referral due to clinicians related barrier	0.54 (0.28–1.04)	0.47 (0.23–0.95)
Late referral from family and patient-related issues	0.29 (0.13–0.69)	0.29 (0.12–0.73)
Late referral due to physicians’ perception of palliative care	0.30 (0.13–0.67)	-

*Abbreviation:* COR Crude odds ratio, AOR, Adjusted odds ratio CI Confidence interval

## Discussions

In this study, we investigated the physician’s referral practices and perceived barriers to timely referrals at the main referral centre in Ghana. The need for palliative care services is typically communicated to patients and their significant others through compassionate discussions led by healthcare professionals. These discussions emphasize the importance of holistic support, pain management, and improving quality of life during serious illness. Patient-centred communication ensures understanding, empathy, and collaboration in decision-making regarding palliative care options. This helps to develop mitigating strategies since early palliative care improves the quality of life and survival and reduces in-hospital deaths, emergency room visits, and hospitalizations in the last phase of life [28–30]. Our study showed that despite having positive perspectives about palliative care services, many of the physicians (68%) attributed late referral practices to their making. This resonates with the study by Kalies et al. [31] and Sorensen et al. [32], who asserted that physicians’ beliefs about palliative care were inconsistent with their practice. The study results also showed that 3.9% of clinicians attributed their late referral practice to the absence of a palliative care specialist in their department. Thus, although palliative care services were available in the hospital, a small fraction of the physicians had not acquainted themselves with the unit. This finding is contrary to a similar study by Ofosu Poku et al. [33] in which few of the clinicians attributed late referrals to the absence of a palliative care specialist in the health facility. The study results also highlight differences between referral practices among physicians. Previous studies suggest longer years of practice positively influence early referral practices, however, our study results showed otherwise. In this study, physicians who had more than three years of medical practice engaged in late referrals practices as compared to those with less than three years of medical practice [34]. Unlike other studies, neither gender nor age of physicians had any influence on the timing of palliative care. Other studies have investigated healthcare professionals’ attitudes and perceptions towards palliative care and referral to palliative care specialists. A study conducted in the United States found that healthcare professionals perceived a lack of knowledge and skills as a barrier to referring patients to palliative care [24, 35]. Meanwhile, Lalani and Cai [36] disclosed that medical practitioners believed that a hurdle, especially in rural areas, was the lack of proper access to palliative care treatments. In a similar vein, medical practitioners believed that a major obstacle was the absence of palliative care services [37]. Nonetheless, this study also discovered that physicians were more likely to recommend patients to palliative care specialists if they had prior training and experience in the field similar to Artioli et al. [38] and Sorensen et al. [39]. Similar to the findings of the current study, Ramos-Vera et al. [40] discovered that medical practitioners considered patient and family resistance as a major obstacle to referral to palliative care. In other words, when medical practitioners believed a patient had a limited life expectancy or that the patient’s symptoms were difficult to treat, they were more likely to refer the patient to palliative care [41, 42]. The results of this study generally agree with earlier studies that found that a barrier to referral is the lack of availability and access to palliative care services. The current study does, however, also raise the possibility that patient and family attitudes regarding palliative care may represent a significant referral barrier, which should be addressed by providing healthcare personnel with support and education.

A basic misunderstanding of the concepts and objectives of palliative care as reported by participants by suggesting that referring a patient to a palliative care professional is equivalent to abandoning them is a flaw. This finding implies that physicians may not have received adequate training in palliative care. Palliative care may not have been adequately taught to many medical professionals during their training, which has resulted in misunderstandings regarding its importance in patient care as indicated by Tanzi et al. [43]. It is significant to note that palliative care is not the same as end-of-life care or abandonment as reported by Johnson et al. and Streeck et al. [44, 45]; rather, it is a way of helping patients with life-threatening illnesses live better by managing their symptoms, offering psychological support, and helping them make decisions. This result emphasizes the need for physicians to be better informed about palliative care. Physicians need to be apprised that bringing a patient to the attention of a palliative care specialist does not imply giving up on them; rather, it means offering all-encompassing, holistic treatment that is aimed at reducing suffering and enhancing the patient’s quality of life in general. Targeted teaching and training initiatives within the medical and nursing curriculum as well as continuous professional development programs are needed to address this misperception to offer patients the best possible care.

### Limitations

The study lists numerous limitations. First of all, because cross-sectional studies only record data at one particular moment in time, it might be difficult to determine if referral patterns and obstacles to palliative treatment have changed over time. Again, because a convenience sample strategy was used, selection may have affected the study, which could have affected the findings’ generalizability. Participants may also experience difficulties accurately recalling information, which could result in biases in reported referral practices or felt barriers to receiving palliative treatment. Furthermore, response bias could be introduced by depending too much on self-reported data, leading individuals to either overreport or underreport their experiences with referral procedures and perceived barriers to palliative treatment. Future research should think about conducting a qualitative study on the topic as there may be a lack of rich qualitative data that might offer deeper insights into the experiences and perspectives of stakeholders involved in palliative care.

## Conclusion

In conclusion, our study revealed a prevalent issue of late referrals to palliative care, with 68.0% attributed to clinician-related barriers. The misconception that referring to a palliative care specialist implies abandonment (92.8%) emerged as a major perceived barrier. This highlights a critical need for targeted interventions addressing physicians’ understanding and perceptions of palliative care. The study further identified significant associations between late referrals and department, religious affiliation, years of practice, and issues related to family and patients. Comprehensive strategies should be devised to enhance medical education, focusing on palliative care principles, to ultimately improve the quality and timeliness of referrals.

### Electronic supplementary material

Below is the link to the electronic supplementary material.

Supplementary Material 1

## Data Availability

All data generated or analyzed during this study are included in this published article.
