# Supplementary material for: Unlocking timely palliative care: assessing referral practices and barriers at a ghanaian teaching hospital
Source: BMC Palliat Care. 2024 Apr 5;23:90. doi: 10.1186/s12904-024-01411-9 (PMC10996152; doi:10.1186/s12904-024-01411-9)
Supplement: Supplementary file 1 — Supplementary Material 1 [file 12904_2024_1411_MOESM1_ESM.pdf]

STRUCTURE QUESTIONNAIRE

1. **Gender:**
   Female [ ]   Male [ ]
2. **Department:**
   Obstetrics and Gynecology [ ]   Child Health [ ]
   Surgery [ ]   Cardiothoracic [ ]
   Medical [ ]   Hematology [ ]
3. **Years of practice:**
   <3 years [ ]   3 years and more [ ]
4. **Rank:**
   House Officer [ ]   Resident [ ]
   Medical Officer [ ]   Consultant [ ]
5. **Religion:**
   Christianity [ ]   Traditional [ ]
   Islam [ ]   Others [ ]
6. **Age:**
   30 years [ ] 31-45 years [ ] 46-50 years [ ] 50 years and above [ ]
7. Late referral are physician-related   Yes [ ] No [ ]
8. Late referral are family and patient-related issues   Yes [ ] No [ ]
9. Late referrals are due to physician perception of palliative care   Yes [ ] No [ ]

BARRIERS TO PALLIATIVE CARE

**Perceived physician-related barriers**				
**No**	**Items**	**Yes**	**No**	**Don't know**
1	Palliative Care specialists are not available in my hospital			
2	Appointments with palliative care specialist are hard to get			
3	My patients or family members do not like being referred to palliative care.			
4	Referring to palliative care specialist means that I abandon my patient			
5	Palliative care specialists discourage active oncological therapy			
6	Palliative care specialists in my country are not experienced/ trained enough.			
7	I can provide better symptom control management than them.			
8	Patient deteriorates faster			
9	Long standing relationship with patient			
10	Poor relationship with palliative care specialist			
